# *Sox9* is critical for suppression of neurogenesis but not initiation of gliogenesis in the cerebellum

**DOI:** 10.1186/s13041-015-0115-0

**Published:** 2015-04-12

**Authors:** Keng Ioi Vong, Crystal Kit Ying Leung, Richard R Behringer, Kin Ming Kwan

**Affiliations:** School of Life Sciences, The Chinese University of Hong Kong, Hong Kong, P.R. China; RGC-AoE Centre for Organelle Biogenesis and Function, The Chinese University of Hong Kong, Hong Kong, P.R. China; Partner State Key Laboratory of Agrobiotechnology (CUHK), The Chinese University of Hong Kong, Hong Kong, P.R. China; Department of Genetics, The University of Texas MD Anderson Cancer Center, Houston, TX 77030 USA

**Keywords:** Sox9, Neural stem cell, Neurogenesis, Gliogenesis, Cerebellum

## Abstract

**Background:**

The high mobility group (HMG) family transcription factor *Sox9* is critical for induction and maintenance of neural stem cell pool in the central nervous system (CNS). In the spinal cord and retina, *Sox9* is also the master regulator that defines glial fate choice by mediating the neurogenic-to-gliogenic fate switch. On the other hand, the genetic repertoire governing the maintenance and fate decision of neural progenitor pool in the cerebellum has remained elusive.

**Results:**

By employing the Cre/loxP strategy, we specifically inactivated *Sox9* in the mouse cerebellum. Unexpectedly, the self-renewal capacity and multipotency of neural progenitors at the cerebellar ventricular zone (VZ) were not perturbed upon *Sox9* ablation. Instead, the mutants exhibited an increased number of VZ-derived neurons including Purkinje cells and GABAergic interneurons. Simultaneously, we observed continuous neurogenesis from Sox9-null VZ at late gestation, when normally neurogenesis ceases to occur and gives way for gliogenesis. Surprisingly, glial cell specification was not affected upon Sox9 ablation.

**Conclusion:**

Our findings suggest *Sox9* may mediate the neurogenic-to-gliogenic fate switch in mouse cerebellum by modulating the termination of neurogenesis, and therefore indicate a functional discrepancy of *Sox9* between the development of cerebellum and other major neural tissues.

## Background

During vertebrate CNS development, the radial glial cells are self-renewing multipotent neural stem cells that generate the major differentiated cell types in the mature brain, including neurons, myelin-forming oligodendrocytes and non-myelinating astrocytes [1]. To construct the remarkable cellular diversity in the CNS, cell fate decisions must be orchestrated by sophisticated networks of signaling molecules and transcription factors; while the combinatorial effects of which tightly regulate cell lineage specification and differentiation within the neural progenitor pool in a spatial-temporal manner. Any defects during the developmental process are therefore detrimental and can lead to various neurological and psychiatric disorders [2,3].

In the developing cerebellum, radial glial-like neural progenitors at the ventricular zone (VZ) are capable of generating the entire repertoire of GABAergic neuronal subtypes and glial cells [4]. Since the onset of cerebellar neurogenesis at embryonic day 10.5 (E10.5), GABAergic neurons including Purkinje cells and deep cerebellar nuclei neurons are produced from the VZ by asymmetric, neurogenic division of radial glial cells and become terminally differentiated [5], whilst neuronal progenitors committed to inhibitory interneurons delaminate from the VZ and continue to proliferate within the prospective white matter (PWM) until after birth [6,7]. During these initial progressive waves of neurogenesis the VZ generates very few, if any, glial populations. Towards late gestation, however, neurogenesis in the cerebellar VZ progenitor pool gradually ceases and is taken over by the production of oligodendrocytes and astrocytes [8]. Indeed, this sequential production of neurons and glia from the VZ neuroepithelium is not a phenomenon unique to the cerebellum, but is rather a process fundamental to the entire developing CNS and is conserved throughout all the vertebrate species [9-12]. Despite the relatively well characterized molecular events leading to stem cell fate decision in the neocortex and spinal cord, analogous mechanisms in the cerebellum that govern the induction of stem cell characteristics and neurogenic to gliogenic fate switch thus the commitment to gliogenesis have remained unclear.

Sox9 belongs to the SoxE subgroup of the highly conserved family of high mobility group (HMG) transcription factors. Over the past two decades, *Sox9* has been shown to govern developmental activities and was implicated in the maintenance of stem cell pool in a wide array of tissues such as testes, pancreas, heart and the skeleton, among others [13,14]. Importantly, genetic analyses of different models have shown that *Sox9* is vital in triggering the switch from neurogenic to gliogenic program at the germinal zones of different neural tissues [10,12]. In the absence of *Sox9*, the spinal cord exhibited a transient increase in the number of motor neurons at the expense of the astroglial progenitor population [12], while mosaic depletion of *Sox9* in the developing mouse retina resulted in agenesis of the Müller glial cells [10]. Noteworthily, *Sox9* expression in the neural stem cells at different regions of the CNS was demarcated for a long time. But it is only until recently, *Sox9* was demonstrated to be indispensable for the establishment and maintenance of neural stem cells in both embryonic and adult CNS [15,16]. In developing cerebellum, *Sox9* is expressed specifically along the VZ where the radial glial cells populate. Given the importance of *Sox9* in several critical aspects of CNS development and that its expression in the cerebellum coincides with robust neurogenic activities, we sought to determine whether *Sox9* is involved in the establishment and maintenance of neural progenitor identity and characteristic, as well as the initiation of gliogenesis in the cerebellum.

Here in this study, using the *Cre/*loxP recombination approach, we specifically ablated *Sox9* expression in the cerebellum by employing a *Sox9* conditional (*floxed*) allele together with either the *Engrailed1 (En1)-Cre* or *Paired box 2 (Pax2)-Cre* allele. Surprisingly, self-renewal capacity of the VZ progenitors was not abolished upon *Sox9* inactivation. Meanwhile, initial neurogenesis at neither cerebellar VZ nor the anterior rhombic lip was not perturbed despite robust *Sox9* expression at these two cerebellar germination zones. Interestingly, generation of Purkinje cells and GABAergic interneurons from the VZ of *Sox9-*null cerebellum increased remarkably was observed in the *Sox9* null cerebellum, the surplus of which probably arise at late gestation when normally neurogenesis should decline. To our surprise, specification of the glial lineage appeared normal in our *Sox9* mutants. These findings suggest that *Sox9* is dispensable for neural stem cell activity and defining the glial fate of these progenitors in the embryonic cerebellum, in contrast to our current understanding of *Sox9* function in other regions of the CNS. However, our data suggests a critical role of *Sox9* in termination of neurogenesis and mediating the complete neurogenic-to-gliogenic fate switch of cerebellar neural progenitors.

## Results

### Sox9 expression in the neural progenitors and glial lineage of cerebellum

**Figure 1:**
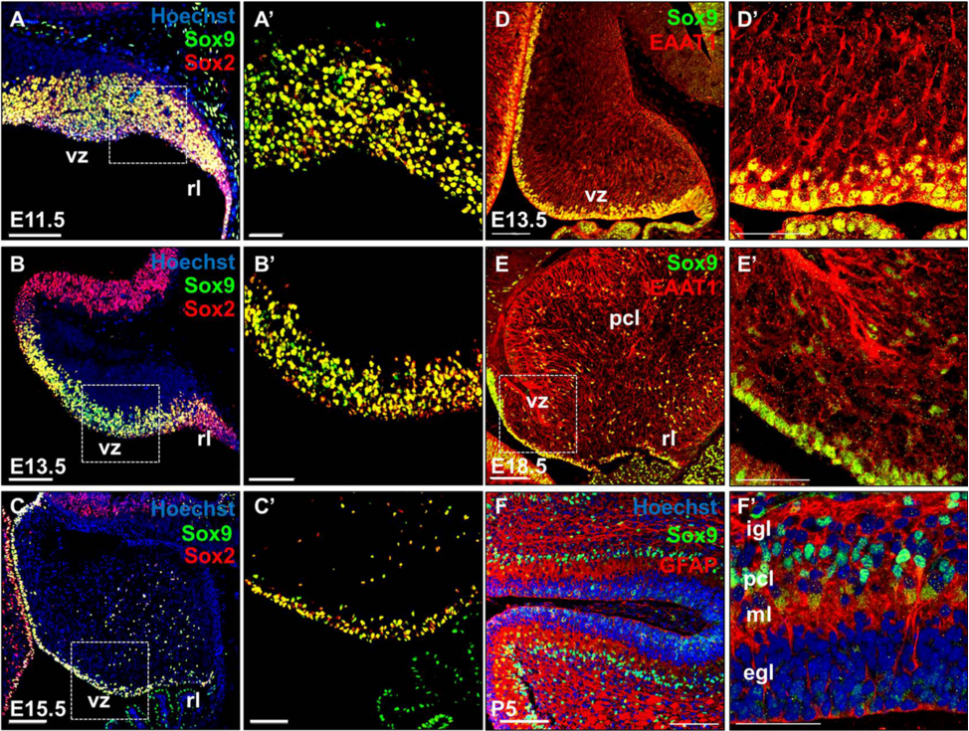
Sox9 expression coincides with markers for neural progenitors, RGCs and Bergmann glia during mouse cerebellum development as revealed by immunofluorescence staining of cerebellar sagittal sections from E11.5 to P5. **(A, A’)** At E11.5, Sox9 immunoreactivity could be observed in the entire cerebellar primordium, covering the newly specified VZ and RL. **(B, B’)** At E13.5, Sox9 expression became restricted to the VZ, while traces of Sox9+ cells were also found in the RL. **(C, C’)** Sox9 expression persisted throughout gestation at the VZ. From E15.5 onwards, Sox9 could also be detected in cells that had delaminated from the VZ as they migrate to the Purkinje cell plate at E18.5. **(A’-C’)** Magnified view of the corresponding VZ region marked by dotted square in **A-C**. Almost all the Sox9 expressing cells were immmunoreactive for the neural stem cell marker Sox2 throughout the embryonic cerebellum development. (**D** and **E**) Co-localization of Sox9 with the glial marker was observed from E13.5 to E18.5. **(D’)** At E13.5, EAAT1 was predominantly localized to the VZ where the Sox9+ radial glial cells reside. **(E’)** After the onset of astrogliogenesis, the majority of EAAT1 expression at E18.5 was found at the PCL where the Bergmann glial cells locate. (**F** and **F’**) By P5, Sox9 signal was found predominantly at the PCL, where it labeled the soma of GFAP^+^ Bergmann glial cells. Abbreviations: egl, external granular layer; igl, internal granular layer; pcl, Purkinje cell layer; ml, molecular layer; rl, rhombic lip; vz, ventricular zone. Scale bars: A-F, 100 μm; A’-F’, 50 μm.

To understand the role of *Sox9* in cerebellar neural progenitor cell fate specification and differentiation, we first performed a detailed analysis of Sox9 protein expression profile during embryonic cerebellar development in mouse. Shortly after neural tube closure and formation of the pontine flexure, the cerebellar primordium is established from rhombomere 1 of the hindbrain between embryonic day (E) 9.5 and E11.5, when the two primary germination zones of cerebellum, the VZ and the anterior rhombic lip (RL) are compartmentalized [17]. Consistent with previous studies in the telencephalon [16], we did not detect any Sox9 expression in the cerebellum before E10.5 (data not shown). At E11.5 however, Sox9 became readily detectable throughout the cerebellar primordium (Figure 1A). Double immunohistochemistry was performed and the vast majority of Sox9 expression in the two cerebellar germination zones coincided with that of Sox2, a marker for actively proliferating neural stem cells and progenitors [18] (Figure 1A’). From E13.5 onwards, Sox9 became restricted to the VZ neuroepithelium (Figure 1B). Thereafter, we still observed close association of Sox9 expression with that of Sox2 (Figure 1B’), while robust expression of Sox9 persisted at the VZ throughout gestation. From E15.5 onwards, some of the Sox9-expressing cells had delaminated from the VZ and were migrating upwards radially towards the presumptive cerebellar cortex (Figure 1C and C’). During cerebellum development, Sox9-expressing cells at the VZ were also immunoreactive for the excitatory amino acid transporter EAAT1 [19] which is an attribute suggestive of the multipotent radial glial cells (Figure 1D). By E18.5, Sox9 expression was detected in the nascent Purkinje cell layer (PCL) where they co-localized with EAAT1, a marker for the differentiated astrocytic lineage at late embryonic stages. On the other hand, Sox9+ cells scattered throughout the prospective white matter (PWM) (Figure 1E). By P5, as the VZ neuroepithelium gradually diminished, Sox9-expressing cells became predominantly situated in the PCL where they also expressed the Bergmann glia-specific glial fibrillary acidic protein (GFAP) consistently (Figure 1F).

### Conditional inactivation of *Sox9* in the mouse cerebellum

**Figure 2:**
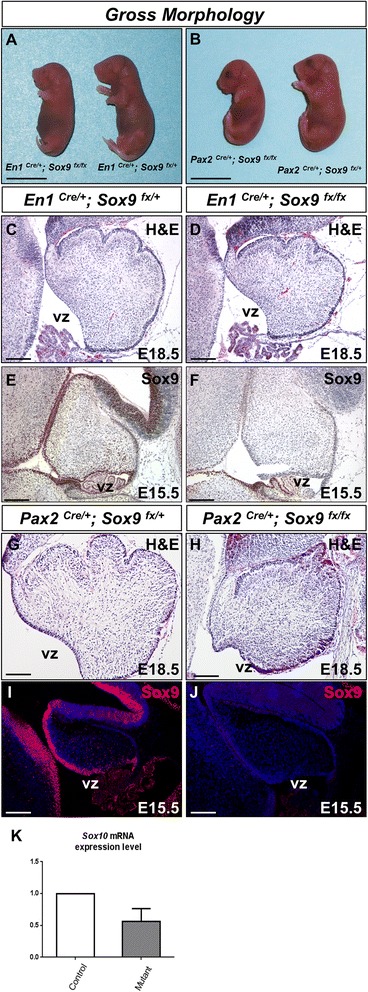
*En1-Cre* and *Pax2-Cre* mediated inactivation of *Sox9* in mouse cerebellum. **(A** and **B)** Conditional knockout of *Sox9* driven by either *En1-Cre* or *Pax2-Cre* resulted in lethality at birth. Gross morphology of E18.5 embryos comparing the control and *Sox9* mutants displayed. **(C** and **D)** H&E-stained cerebellar sagittal sections showing no alterations in morphological criteria of the cerebellum upon *En1-Cre* mediated *Sox9* inactivation, despite efficient ablation of *Sox9* expression in the mutant cerebellum as shown by the immunohistochemical staining detecting Sox9 indicated in **(E** and **F)**. Strong expression of Sox9 could be observed in the control along the VZ at E15.5 while the mutant cerebellum lacked any Sox9 immunoreactivity. **(G** and **H)** Defect in cerebellar foliations was observed at E18.5 in the *Pax2-Cre Sox9* mutant. **(I** and **J)** The fidelity of *Pax2-Cre* mediated *Sox9* inactivation was validated by the complete absence of *Sox9* expression in the mutant cerebellum. **(K)** Upon ablation of *Sox9* using *Pax2-Cre,* no significant up-regulation of the closely related SoxE family member *Sox10* transcript expression level was observed in the mutant using quantitative real-time PCR (*p =* 0.1617). The error bars indicate standard deviations. Abbreviations: vz, ventricular zone;. Scale bars: A and B: 1 cm; C-J: 100 μm.

Having observed that Sox9 was robustly expressed in the neural progenitors and differentiated glial cells throughout cerebellum development, we hypothesized that Sox9 may regulate the cerebellar neural progenitor pool and their fate commitment to gliogenesis. To address the role of Sox9 during cerebellum development, Sox9 loss-of-function analysis in the mouse cerebellum was performed. To circumvent the early embryonic lethality owing to cardiac neural crest defects [20] that precludes the assessment of cerebellum development, we employed *En1* or *Pax2* driven Cre, both of which possess robust activity at the mid-hindbrain boundary of the early neural tube to ensure a complete knockout of *Sox9* in the cerebellum [21-23]. Conditional knockout of *Sox9* driven by either *En1-Cre* or *Pax2-Cre* resulted in lethality very shortly after birth (Figure 2A and B). Conditional inactivation of *Sox9* with *En1-Cre* in the cerebellum did not affect the morphological criteria of cerebellar development as revealed by general histological examination with hematoxylin and eosin (H & E) staining (Figure 2C and D). However, *Sox9* deletion mediated by *Pax2-Cre* resulted in improper or obscure cerebellar foliation noticeable from E16.5 onwards, the severity of which was variable between litters (Figure 2G and H). In these mutants, Cre expression in the cerebellar primordium efficiently ablated *Sox9* in all cerebellar cells and their derivatives, as evident by PCR analysis with cerebellar genomic DNA (data not shown) and the complete absence of Sox9 immunoreactivity in the cerebellum of the *Sox9* mutants at all developmental stages being investigated, thus confirming the fidelity of *En1-Cre* and *Pax2-Cre* mediated functional inactivation of *Sox9* (Figure 2E,F,I and J). Sox8 and Sox10, the other members of the SoxE family, are also expressed in the radial glia, astrocytes and oligodendrocyte progenitors of the CNS [24]. Given that Sox8 or Sox10 can partially compensate for the loss of *Sox9* in the spinal cord and telencephalon respectively [12,16], the expression of *Sox8* and *Sox10* in our *Sox9* mutants was also examined. Sox8 transcripts were not detected in the cerebellum (data not shown) while *Sox10* transcripts were only scarcely expressed as compared to *Sox9* mRNA expression. Nonetheless, *Sox10* mRNA expression was not up-regulated in *Sox9* null cerebellum as indicated by quantitative real-time PCR (Figure 2K).

### Sox9 was dispensable for self-renewal capacity of cerebellar neural progenitors

**Figure 3:**
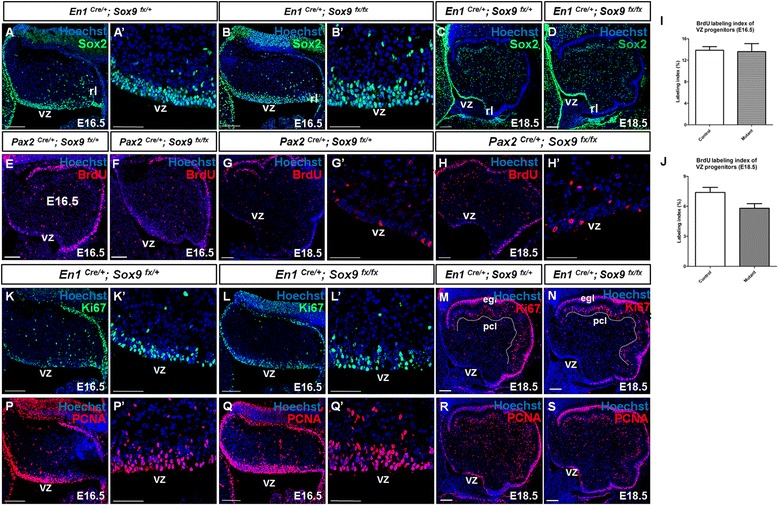
Self-renewal of neural progenitors in cerebellum was not abrogated upon *Sox9* inactivation as revealed by immunofluorescence staining on cerebellar sagittal sections. **(A-D)** Expression of the neural stem cell marker Sox2 was not lost in the *Sox9-*null cerebellum during late gestation under both *En1-Cre* and *Pax2-Cre* mediated *Sox9* inactivation. In both groups, scattered signals were observed in the VZ and the prospective white matter, while the EGL displayed the strongest signal which corresponded to the actively proliferating granule cell precursors. **(A’)** and **(B’)** showed enlarged view of the VZ. **(E-J)** Quantification of mitotic activity at the VZ by double pulses of BrdU at either E16.5 **(E-F)** or E18.5 **(G-H)** did not reveal any difference in the proliferation of VZ neural progenitors between control and the *Pax2-Cre* induced *Sox9*-null mutant. The number of BrdU-labeled cells in the VZ was divided by the total number of VZ cells to obtain the labeling index **(I-J)**. No statistically significant difference was observed using the Student’s *t*-test at both E16.5 (I; *p* = 0.4906) and E18.5 (J; *p* = 0.1633). **(K-R)** In addition, we did not observe any alterations in the expression of proliferation associated markers PCNA and Ki67 upon *Sox9* inactivation. **(A’-Q’)** are the enlarged view at the VZ of **(A-Q)**. The error bars indicate standard deviations. Abbreviations: egl, external granular layer; pcl, Purkinje cell layer; rl, rhombic lip; vz, ventricular zone. Scale bars: **A-S**: 100 μm; A’-B’, G’-H’, K’-L’, P’-Q’: 50 μm.

To determine whether the neural stem cell identity of the VZ neural progenitors was perturbed upon *Sox9* ablation, expression pattern of the neural stem cell marker Sox2 was examined by immunostaining. Interestingly, Sox2 expression was retained in the VZ and PWM of the *Sox9*-null cerebellum even at late gestation period between E16.5 and E18.5 (Figure 3A-D). We then asked whether the self-renewal capacity of the VZ neural progenitors was abrogated in the *Sox9* mutants. To assess the mitotic index of VZ progenitors, bromodeoxyuridine (BrdU) incorporation efficiency was examined. Pregnant female mice at E16.5 or E18.5 of gestation were injected with BrdU twice at 30-minute intervals and sacrificed. We did not observe any statistically significant difference in the proportion of BrdU incorporated neural stem cells within VZ of controls and *Sox9* mutants at both stages, while the spatial distribution of BrdU+ cells in *Sox9-*null cerebellum also appeared normal (Figure 3E-J). In addition, expression of the proliferating cell nuclear antigen (PCNA) and Ki67 which denotes mitotic activity in the *Sox9* mutant cerebella were not impaired as well throughout the course of cerebellar development (Figure 3K-R). We also examined the possibility that loss of *Sox9* may lead to increased programmed cell death in the mutant VZ by detecting cleaved caspase 3 signals, a hallmark for apoptosis, by immunostaining. Again, few cells positive for cleaved caspase 3 was observed in the WT or *Sox9-*null cerebellum, indicating that no apoptotic activity among the cerebellar progenitors was up-regulated upon *Sox9* inactivation (data not shown). Together, these data suggest that *Sox9* is not a critical regulator that governs the self-renewal and survival of neural progenitors in the developing cerebellum.

### Sox9 was not required for the neuronal differentiation of cerebellar progenitors

**Figure 4:**
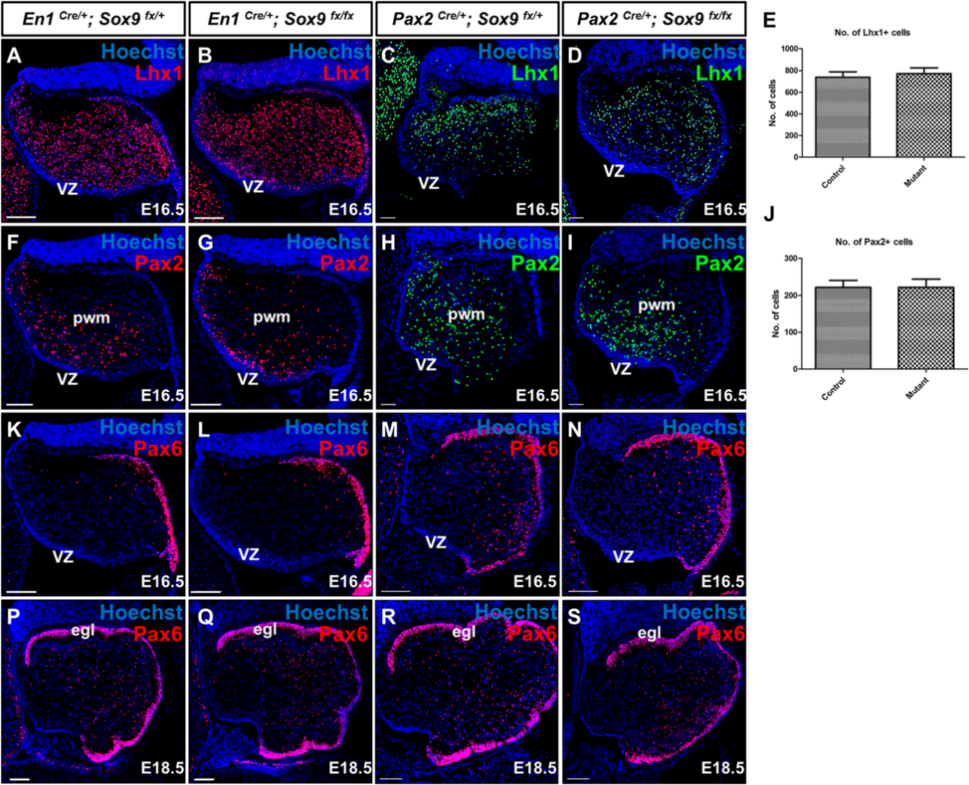
Cerebellar neural stem cell multipotency was not abolished in *Sox9-*null cerebellum under both *En1-Cre* and *Pax2-Cre* mediated *Sox9* inactivation as shown by immunofluorescence staining on cerebellar sagittal sections. **(A-D, F-I)** No defects in the neurogenesis of VZ-derivatives including Purkinje cells (PCs) and GABAergic interneurons was observed, as indicated by the normal distribution of Lhx1^+^ PCs **(A-D)** and Pax2^+^ interneurons **(F-I)** between control and *Sox9* mutant cerebella at E16.5. Both the number of Lhx1+ PCs and Pax2+ interneurons appeared normal in the *Sox9* mutants (**E**; *p* = 0.6870 and **J**; *p* = 0.9980). **(K-S)** Specification of the RL-derived progenitors committed to become granule cells was also normal, as exemplified by robust expression of Pax6 at the EGL. The differentiation of progenitors into different neuronal subtypes suggests that the multipotency of neural stem cells in cerebellum was retained upon *Sox9* inactivation. Abbreviations: egl, external granular layer; pwm, prospective white matter; vz, ventricular zone. Scale bar: 100 μm.

Previous studies in forebrain have suggested that *Sox9* is critical for differentiation of neural stem cells into mature neurons as evident by a drastic loss of Tuj1 expression in the dorsal telencephalon and spinal cord of the *Sox9* mutant [16]. We therefore assessed the multipotency of *Sox9-*null VZ progenitors in generating different neuronal subtypes. Multipotent RGC-like progenitors in the VZ give rise to all the inhibitory neurons in the cerebellum including Purkinje cells (PCs) and GABAergic interneurons [25]. PCs are recognized by the expression of the LIM-homeodomain transcription factor Lhx1 [26], the calcium-binding protein Calbindin D-28K [27], or the forkhead domain transcription factor Foxp2 at late embryonic stage [28]. On the other hand, the paired box transcription factor Pax2, labels postmitotic GABAergic interneurons or their progenitors undergoing the final mitotic cycle [7]. Unexpectedly, immunostaining at E16.5 revealed no significant difference in neurogenesis of either PCs or GABAergic interneurons (E and J), in both the control and *Sox9* mutant cerebella as exemplified by the normal expression pattern of both Lhx1 (Figure 4A-D) and Pax2 (Figure 4F-I). Similarly, the loss of *Sox9* had no observable effect on the general localization and differentiation of RL derivatives, including the Pax6-expressing excitatory granule cells (Figure 4K-S), despite *Sox9* expression in the RL progenitors during the phase of granule cell specification. These results support that, unlike in the dorsal telencephalon, subventricular zone as well as the spinal cord [12,16], Sox9 is dispensable for the maintenance of neural progenitor multipotency in the cerebellum.

### Prolonged and enhanced neurogenesis in the *Sox9-*null cerebellum

**Figure 5:**
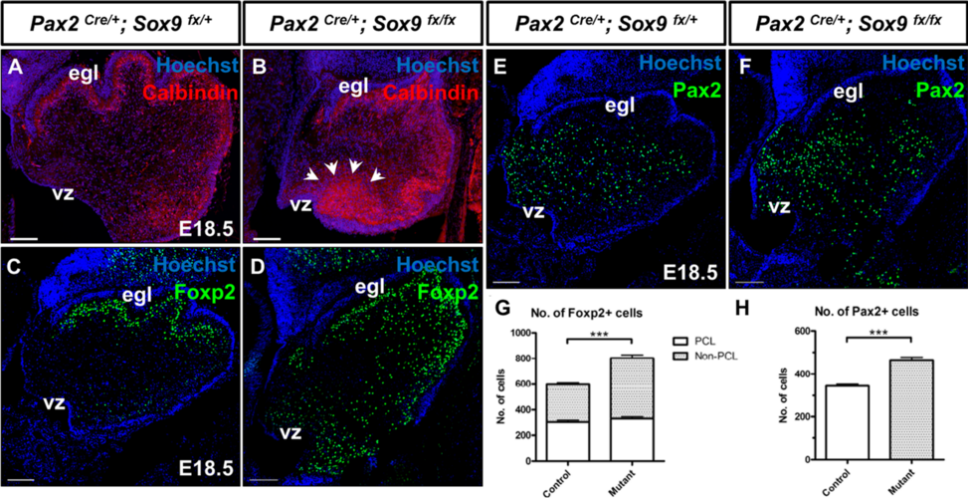
Increased neurogenesis of VZ-derivatives in *Sox9-*null cerebellum. **(A-B)** Immunofluorescence staining on cerebellar sagittal sections showed an up-regulation of Calbindin expression in the *Sox9* mutant with an ectopic expression domain close to the VZ (arrows). **(C-D)** More Foxp2^+^ cells could be found in the *Sox9* mutant cerebellum and the spatial distribution of which closely mimicked the expression pattern of Calbindin (compared with **B**). **(G)** Quantification of Foxp2^+^ cells confirmed a 1.3 fold increase in the Purkinje cell populations, the surplus of which was predominantly localized outside the PCL at the vicinity of VZ. **(E, F** and **H)** The number of Pax2-expressing cells was also increased by about 30% in the *Sox9*-null cerebellum than in the control. Asterisks (***) indicate statistically significant difference of *p*-value < 0.0001 by the Student’s *t*-test. The error bars indicate standard deviations. Abbreviations: egl, external granular layer; vz, ventricular zone. Scale bars: A-C: 100 μm.

Having observed no significant decrease in the mitotic activity among the VZ progenitors upon *Sox9* ablation, we further sought to determine whether neurogenesis at the VZ was defective. Postmitotic PCs are generated from VZ between E11.5 and E13.5, and subsequently migrate along the radial glial fiber to form the Purkinje cell layer (PCL) where they undergo extensive morphological remodeling [29,30]. Shortly before birth at E18.5, the *Sox9* mutant cerebellum exhibited an expanded expression domain of Calbindin localized ectopically close to the VZ, in addition to its normal expression at the PCL (Figure 5A-B). This leads to our speculation that neurogenesis for PCs was enhanced upon *Sox9* knockout. Since Calbindin is a cytoplasmic protein and renders validation of this hypothesis difficult, we therefore analyzed PCs generation by quantifying Foxp2-expressing cells at E18.5. While Foxp2 immunoreactivity was observed predominantly in the PCL of the control, many ectopic Foxp2-expressing cells could be found in the vicinity of the VZ in the *Sox9-*null cerebellum, faithfully recapitulating the expression pattern of Calbindin (Figure 5C-D). A 1.3 fold increase in the total number of Foxp2-expressing cells was observed in the mutant cerebellum (*p* < 0.0001), most of which was contributed by an increase in the Foxp2-expressing cells outside the PCL (Figure 5G). We then determined whether the increased neurogenesis at VZ was consistent throughout different neuronal subtypes. Quantification of Pax2-expressing cells also revealed a 1.3 fold increase (*p* < 0.0001) in the total number of postmitotic GABAergic interneurons in the *Sox9-*null cerebellum (Figure 5E,F and H). Taken together, the ablation of *Sox9* in the cerebellum *in vivo* did not perturb the initial generation of PCs and GABAergic interneurons. Instead, *Sox9* inactivation led to markedly increased neurogenesis from the VZ and subsequently a remarkably enhanced number of these inhibitory neurons.

**Figure 6:**
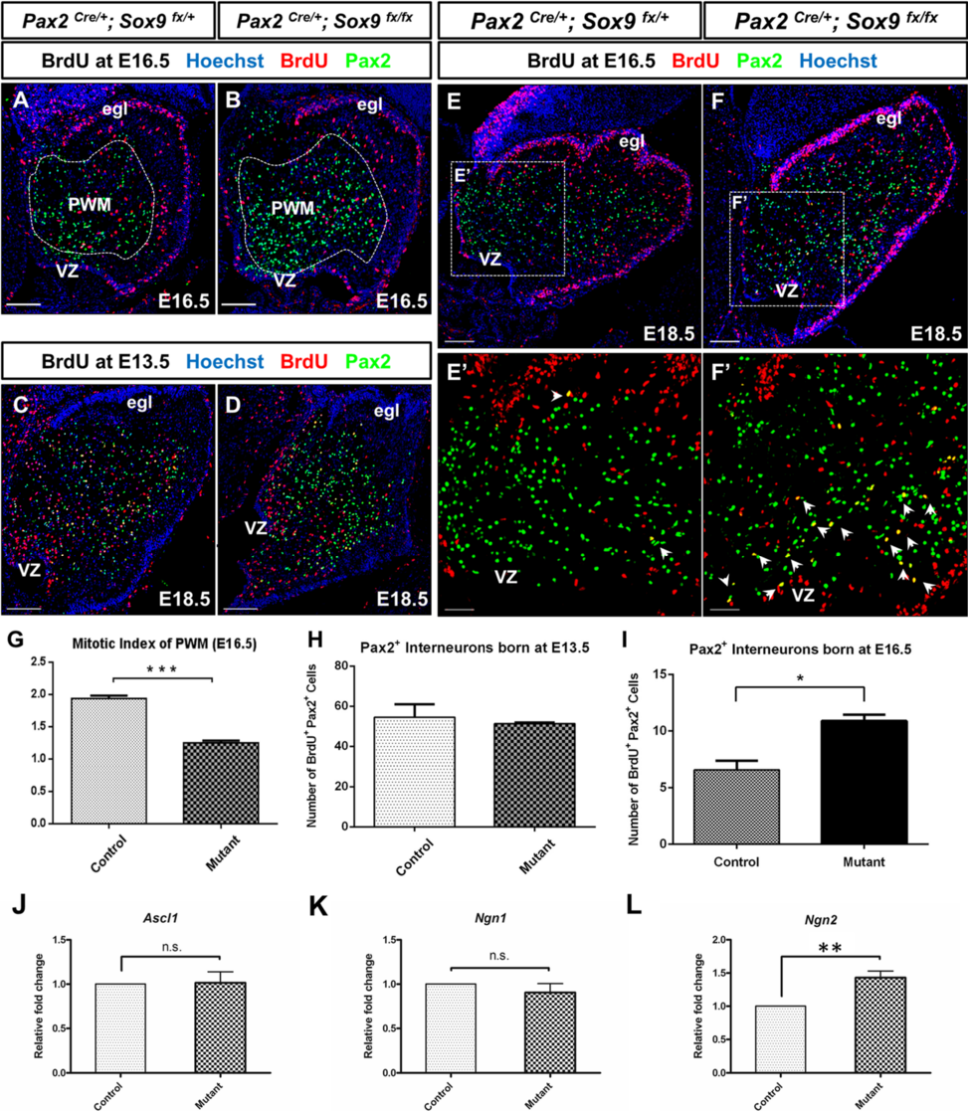
Increased neuronal populations due to prolonged neurogenesis at the VZ as revealed by immunofluorescence staining on cerebellar sagittal sections. **(A, B)** Becoming active since late gestation, the prospective white matter (PWM) (dotted area) is where interneuron progenitors delaminated from the VZ proliferate, gradually fate restricted and become postmitotic. The identity of PWM as a secondary germinal site in the developing cerebellum was exemplified by identifying the BrdU positive cells within the Pax2 positive domain. **(G)** Proliferation within the PWM at E16.5 was reduced in *Sox9-*null cerebellum when compared with control (***, *p* < 0.005). **(C, D **and **H)** BrdU tracing experiments from E13.5 to E18.5 revealed no difference in the number of Pax2+ interneurons born at around E13.5 between controls and mutants. **(E, F **and **I)** In contrast, BrdU tracing experiments from E16.5 to E18.5 showed significantly more interneurons were born at around E16.5, as evident by increased number of double Pax2+ BrdU+ cells localized at the proximity of VZ in *Sox9* mutant cerebellum (E’ and F’, arrows) (*, *p* < 0.05). **(J, K **and **L)** *Ngn2* expression level was more than 1.3 fold higher in E16.5 *Sox9* mutant cerebellum (**, p < 0.01), while that of *Ascl1* and *Ngn1* was not significantly different between controls and mutants (*p =* 0.9072 and *p =* 0.3896 respectively). **E’** and **F’** are magnified view of the area enclosed by inset in **E** and **F**. Abbreviations: egl, external granular layer; n.s., not statistically significant; vz, ventricular zone. The error bars indicate standard deviations. Scale bar: 100 μm.

In contrast to PCs which are postmitotic upon migration from the VZ, GABAergic interneuron progenitors are still proliferative upon delamination from the VZ and subsequently populate a secondary germinal site known as the prospective white matter (PWM) which is active throughout late embryonic and early postnatal development [7]. We therefore questioned whether the surplus of Pax2-expressing cells was a consequence of enhanced proliferating activity within the PWM. To clarify this point, embryos were pulsed with BrdU 30 min prior to sacrifice, and mitotic activity at the PWM was quantified by counting BrdU-labeled cells within the Pax2-expressing domain in the cerebellar cortex. Our results, however, indicated that proliferation at the PWM was reduced by about 35% in the *Sox9-*null cerebellum (*p =* 0.0012) (Figure 6A,B and G), which was also evident by remarkably fewer PCNA- or Ki67- positive cells found within the PWM in *Sox9-*null cerebellum (Figure 3K-R). Thus, the increased neurogenesis of Pax2+ interneurons observed is unlikely due to enhanced proliferative activity at the secondary germinal zone. To determine the birthdate of the surplus of postmitotic Pax2-expressing interneurons found in *Sox9-*null cerebellum at E18.5, control and mutant embryos were pulsed with BrdU at E13.5 or E16.5 and sacrificed at E18.5. In the 5-days BrdU tracing, the number of Pax2/BrdU double positive cells found in control or mutant cerebellum was similar, suggesting that the extent in the generation of GABAergic interneurons at around E13.5 was not significantly up-regulated in the *Sox9* mutants (Figure 6C, D and H). In contrast, the number of cerebellar Pax2-expressing cells born at E16.5 was about 60% more in *Sox9-*null cerebellum compared with the control (Figure 6E, F and I). Since we did not observe any increase in proliferative activity at the PWM, our data therefore suggested that neurogenesis at the VZ after E16 was significantly enhanced upon knockout of *Sox9* in cerebellum. Notably, the majority of Pax2-positive interneurons born at E16.5 in *Sox9* mutants were clustered at the vicinity of VZ, further suggesting that the surplus of GABAergic interneurons were originated from the cerebellar VZ, instead of being the descendants of progenitors within the PWM (Figure 6E’ and F’). The expression of proneural genes at E16.5, when normally the neurogenic-to-gliogenic fate switch is occurring in the cerebellum, was examined by quantitative RT-PCR. The bHLH transcription factor *Ngn2* mRNA was up-regulated by around 30% in the *Sox9-*null cerebellum (*n =* 6, *p* = 0.0070) (Figure 6L); while *Ascl1* and *Ngn1* expression level in *Sox9* mutants did not differ from the controls significantly (Figure 6J and K).

### Glial fate determination in the cerebellum was not impaired upon loss of *Sox9*

**Figure 7:**
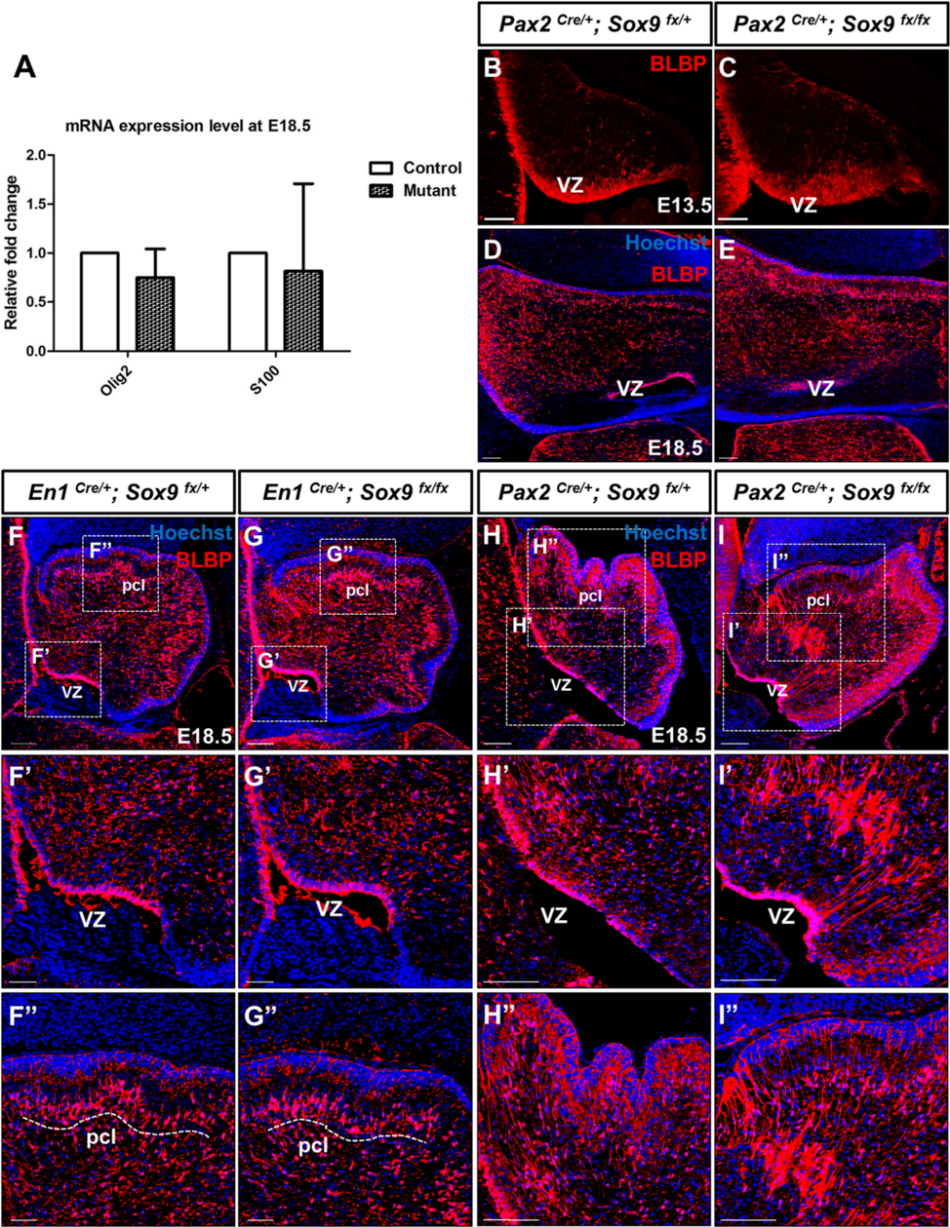
Bergmann glia specification was not abolished in *Sox9-*null cerebellum. **(A)** Real-time PCR of the oligodendrocyte marker Olig2 and astrocyte marker S100 showed that gliogenesis was not perturbed in *Sox9* mutant (*p =* 0.2764 and *p =* 0.7551 respectively). **(B-C)** At E13.5, BLBP expression that marked the early-born radial glial cells which function as the cerebella neural stem cell pool, spanning the entire VZ in both the control and *Sox9* mutant. Expression of BLBP persisted in the cerebellum throughout embryonic development as indicated by both coronal sections **(D-E)** and sagittal sections **(F-I)**. **(F’-I’)** Particularly strong BLBP signal in the VZ where soma of radial glia resides was seen in both control and mutant cerebella. Filamentous BLBP immunoreactivity of the glial scaffold was not disrupted in the *Sox9-*null cerebellum. **(F”-I”)** Robust BLBP expression representing prospective Bergmann glia could be observed in the PCL of *Sox9* mutants (broken line), with the glial endfeet which terminated at the pial surface equally conspicuous in the control and *Sox9* mutant.

**Figure 8:**
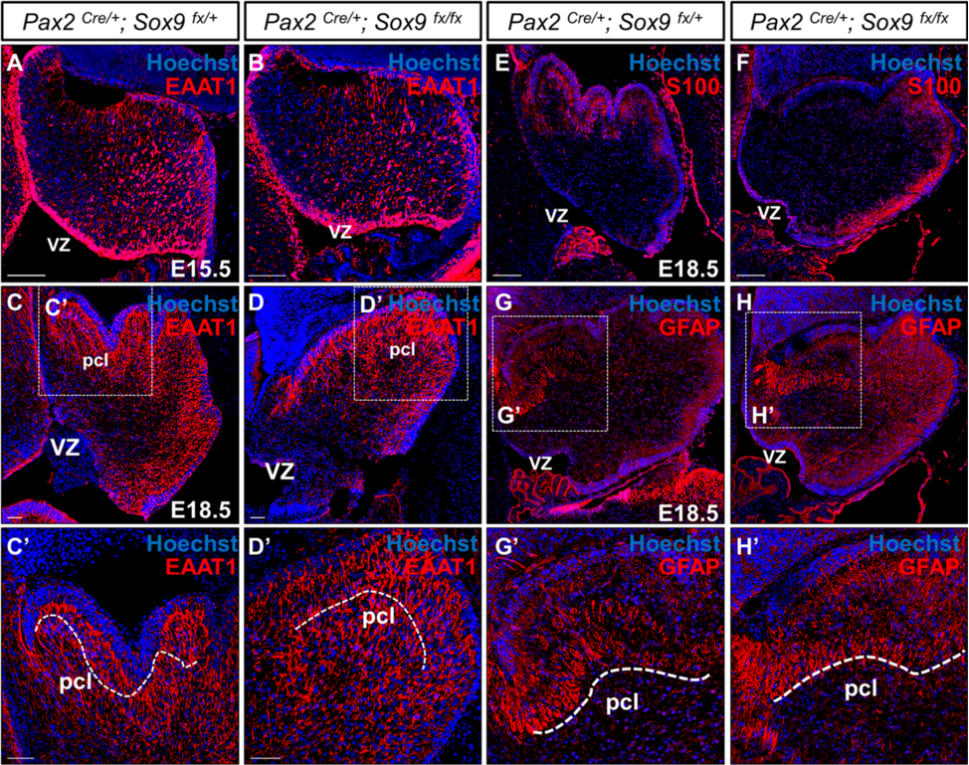
Normal gliogenesis in the *Sox9* mutant cerebellum as shown by immunofluorescence staining on cerebellar sagittal sections. **(A-B)** Robust expression of EAAT1 at E15.5 in both control and *Sox9* null mutants further confirmed the intactness of radial glial scaffold. **(C-D)** At E18.5, EAAT1 expression was found in the PCL, similar to that of BLBP. **(C’-D’)** The glial filaments with EAAT1 immunoreactivity were prominent in the mutants despite inactivation of *Sox9*. **(E-F)** The periventricular expression of S100 at the PCL also suggests the presence of astroglia in both the control and *Sox9* mutant cerebella. **(G-H)** The normal expression of GFAP in the *Sox9* mutant cerebellum suggested that differentiation of Bergmann glia was intact upon knockout of *Sox9*, as evident by the robust distribution of GFAP especially at the PCL where Bergmann glia are found **(G’-H’)**. Abbreviations: pcl, Purkinje cell layer; vz, ventricular zone. Scale bars: 100 μm.

The increase in neuronal population in *Sox9* mutant cerebellum as a consequence of extended neurogenesis during late gestation prompted us to examine whether this phenotype occurred at the expense of gliogenesis which commences during this developmental window. We first studied expression of the oligodendrocyte marker Olig2 and astrocytic marker S100 by quantitative PCR. To our surprise, neither of their expression was significantly diminished in *Sox9* mutants (Figure 7J) We then analyzed the expression of the glial-specific brain lipid-binding protein (BLBP) in histological sections of the control and *Sox9* CKO mutant cerebella. At E13.5, widespread expression of BLBP could be observed at the VZ and its vicinity in both the control and *Sox9* mutant (Figure 7B-C). Unexpectedly, though at E18.5, strong expression of BLBP persisted in both the control and *Sox9-*null cerebellum (Figure 7D-I). BLBP signal could be detected along the entire medial-to-lateral (vermal-to-hemispherical) axis in coronal cerebellar sections of both the controls and mutants, consistent with the observation described above (Figure 7D-E). Robust immunoreactivity for BLBP corresponding to the soma of radial glial cells was also observed in the VZ throughout cerebellum development (Figure 7F’, G’, H’ and I’), further supporting the intactness of the neural stem cell pool even upon loss of *Sox9* in the mutant cerebellum. At this stage, BLBP was also expressed by precursors of the Bergmann glia (BGs) which are cerebellum-specific astrocytes intermingled among the PCs and have the glial processes extending through the molecular layer (ML) with the endfeet terminating at the pial surface [31]. In both the control and *Sox9* mutant cerebellum, distinctive BLBP expression could be observed in the PCL and at the pial surface, indicating the correct specification of BG identity in the absence of *Sox9* (Figure 7F”, G” H” and I”). The general orientation of the glial process arrangement was also not perturbed in the *Sox9* null cerebellum. In addition, glial cells express membrane-bound excitatory amino acid transporters (aka glutamate transporters) to terminate excitatory synaptic transmission by removal or uptake of glutamate from neuronal synapses [32]. In the cerebellum, the excitatory amino acid transporter EAAT1 or GLAST is expressed specifically in the radial glial cells or later in the BGs surrounding the excitatory dendritic spines on PCs [19,33]. At E15.5 when the transdifferentiation of radial glia into BGs was yet initiated, EAAT1 expression was predominantly localized in the VZ and along the radial glial scaffold in the control cerebellar cortex and was not abrogated in *Sox9-*null cerebellum (Figure 8A and B). At E18.5, filamentous pattern of EAAT1 expression was observed in the PCL and pial surface of both the controls and *Sox9* mutants, further strengthening our initial findings that gliogenesis in cerebellum was not affected by the inactivation of *Sox9* (Figure 8C, C’, D and D’). In addition, expression of S100, a marker for mature Bergmann glia and their precursors, was as well not diminished in the PCL of *Sox9-*null cerebellum (Figure 8E and F). We also sought to determine whether the terminal differentiation of Bergmann glia was impaired upon *Sox9* knockout, although this mainly occurs postnatally while our mutants died shortly after birth. The glial fibrillary acidic protein (GFAP) is a defined marker for differentiated Bergmann glia and also white matter fibrous astrocytes [34,35]. At E18.5, GFAP expression was seen at the PCL and some portion of the PWM in both the control and mutants (Figure 8G, G’, H and H’), indicating that astroglial differentiation was not defective, at least until birth, in the *Sox9*-deficient cerebellum.

## Discussion

Despite extensive efforts devoted to the investigations in developmental function of SoxE family transcription factor Sox9 over the past two decades since its initial annotation [36], the first studies that indicated relevant functional roles of Sox9 for neural stem cells appeared only recently [15,16]. Meanwhile, multiple studies have underscored the importance of Sox9 in gliogenesis at different CNS tissues including the retina and spinal cord. In this study, we demonstrated that Sox9 is robustly expressed in the neural progenitors and astrocytes of the developing cerebellum. Through the conditional knockout of *Sox9* in cerebellum, we presented evidence that self-renewal and multipotency of cerebellar neural progenitors did not require Sox9 while normal glial specification was not abolished. Surprisingly, our data suggest that Sox9 is not critical for the aforementioned developmental activities at the cerebellum as in elsewhere of the CNS. Instead, we demonstrated an extended neurogenesis at the *Sox9-*null VZ during the gliogenic period. Our study shall provide novel clues on the diverse roles of Sox9 in neural development, which will be crucial for understanding the cellular and molecular basis underlying the functional discrepancy of Sox9 in different CNS tissues.

### Dispensability of Sox9 in establishing and maintaining neural stem cell identity in the cerebellum

In accordance with previous descriptions of close association between Sox9 and Sox2/EAAT1 expression in the retina [10], spinal cord and dorsal telencephalon of the forebrain [16], we showed that Sox9 was first present in cells of the cerebellar VZ neuroepithelium which display attributes suggestive of multipotent neural progenitor and radial glial cell (RGC) identity, as evident by coincidence of Sox9 with Sox2 or EAAT1 expression throughout the entire period of embryonic cerebellum development. Our results therefore support that the co-expression of Sox9 with Sox2 is a conserved feature observed across the entire developing CNS, and substantiate Sox9 as a defined marker for neural stem cells.

Recently, it has become established that the RGCs represent the neural stem cells (NSCs) of the developing nervous system, rather than merely a specialized type of glial cells playing supportive and scaffolding role as were regarded in the historical perspective [37-39]. RGCs are also widely recognized to possess the potential for generating both neurons and glial cells [1,37]. Provided that Sox9 has been implicated in stem cell maintenance in a large array of tissues including the intestinal epithelium [40], hair follicle [41], pancreas [42] as well as liver [40], and more importantly robust expression of Sox9 in neural progenitor pool throughout the developing CNS, the involvement of Sox9 in the neural stem cell function is therefore a tempting hypothesis. Indeed, a drastic loss of proliferative progenitors and their self-renewal capacity was noted in the dorsal telencephalon and spinal cord as a consequence of inactivating *Sox9* specifically at the RGCs in the entire CNS [16]. The remnants of neural progenitors also fail to differentiate into either mature neurons or macroglia, reflecting the indispensability of Sox9 in ensuring neural progenitor multipotency. Here we attempted to ablate *Sox9* with *En1-Cre* and *Pax2-Cre* driver lines which are expressed early at the mid-hindbrain boundary, well before the initial establishment of RGCs along the VZ neuroepithelium. While the ablation of *Sox9* in the dorsal telencephalon interrupts the establishment of S100-expressing RGCs, our mutants did not exhibit any defects in either the initial induction or maintenance of RGCs. Moreover, normal expression of PCNA, Ki67 and similar efficiency of BrdU incorporation in the mutant cerebellum clearly demonstrated that proliferative activity and self-renewal potential of the cerebellar progenitors was not abrogated or diminished upon *Sox9* inactivation. In addition, differentiated neurons of different subtypes were observed in both the controls and *Sox9* mutants, indicating no defects in the developmental potential of *Sox9-*null cerebellar progenitors. Our results thus vastly contradicts with the current perceived model that Sox9 is a critical transcription factor governing neural stem cell maintenance.

### Sox9-independent astroglial specification in the cerebellum

Since late gestation, Sox9 expression becomes restricted to cells which are glial-committed, differentiated and eventually colonized the PCL as prospective Bergmann glial cells (BGs) which are the most abundant astrocytes found specifically in the cerebellum. Provided that Sox9 is well documented to be involved in gliogenesis during the nervous system development and is expressed persistently in the cerebellar astrocytes, we examined the glial phenotypes in our *Sox9* mutants. Unexpectedly, typical expression pattern of the glial marker BLBP and EAAT1 was evident at the PCL of *Sox9-*null cerebellum, suggesting that fate specification of BGs is normal in the complete absence of Sox9 in the radial glial neural progenitors. By contrast, multiple studies have demonstrated *Sox9* as a key factor determining commitment to glial lineage at the embryonic spinal cord [12]. *Sox9* is also the master regulator triggering the switch from neurogenesis to gliogenesis in both chick and mice by recruiting the nuclear factor-I A [43]. Inactivation of *Sox9* in the retina led to failure in the specification and/or differentiation of Müller glial cells, a specialized type of astrocytes in the retina [10]. The gliogenic nature of Sox9 is also well reflected in the manipulation of the stem cell niche in adult brain, where overexpression of Sox9 at the subventricular zone favors gliogenesis of GFAP-positive astrocytes and simultaneously eliminating neurogenesis completely [15]. With regard to our genetic loss-of-function analysis in the cerebellum, our study has therefore provided new insight on the dispensability of Sox9 in gliogenesis of cerebellum, which challenge the current perception that Sox9 is the indispensable player orchestrating neural stem cell fate decision towards gliogenesis. There may be difference in functional requirement of Sox9 within different tissue types of the CNS. It is important to emphasize that here we argue a dispensable role of *Sox9* in mediating the specification of glial fate rather than in differentiation of BGs *per se*. Since the terminal differentiation of BGs peak postnatally and that our *Sox9* mutants died at birth, the possibility that Sox9 is required for this process should not be precluded despite normal GFAP expression in the mutant cerebellum. Being the specialized type of cerebellar astrocytes, emerging reports are beginning to propose that Bergmann glia actually represents a cohort of adult neural stem cells in the mature cerebellum [44]. Although this hypothesis still awaits substantial evidence, previous study has indicated that Bergmann glia expressed several SOX family transcription factors associated with stem cell maintenance including Sox1, Sox2, Sox3 and Sox9 in the adult cerebellum [45]. Nevertheless, inactivation of *Sox9* in the cerebellum did not elicit any abnormality in the discrete Sox2 expression within the PCL. This suggests that the specification of BG identity is not dependent on Sox9 function, and further strengthening our conclusion that Sox9 is dispensable for stem cell maintenance in the cerebellum.

### Sox9 can suppress neurogenesis in the cerebellum at late gestation

During the establishment of the vertebrate CNS, neurons are born first followed by glial cells [46]. Underlying rationale for this evolutionarily conserved phenomenon is that the position and number of neuron-supporting glial cells can be determined by matching to the neuronal circuitry [47]. Individual neural stem cells (or RGCs) give rise to both neuronal and glial lineages, with the repertoire of progenies changed over time [48-50]. Stem cell fate decision between commitment to neurons or glial cells is therefore critical, in the sense that appropriate interaction between the neuronal circuitry and glial scaffold is a prerequisite for proper CNS functionality [51,52]. As a corollary, failure in generating and organizing the neurons and glia in a balanced proportion is often ensued by detrimental pathological conditions. For example, loss of astrocytes and their EAAT family members may lead to excitotoxicity and is one of the underlying causes of amyotrophic lateral sclerosis [53]. The dysfunction of BG could cause PCs degeneration and contribute to spinocerebellar ataxia (SCA) 7 pathogenesis [54]. On the other hand, the uncontrolled proliferation of glia progenitors result in highly aggressive pediatric brain tumorigenesis [55,56]. Neural stem cell fate determination is therefore tightly orchestrated by intricate interplay between extrinsic environmental cues and intrinsic transcriptional cascades, which together first directs neural progenitors to neurogenesis and later switches the molecular program to gliogenesis [35]. The occurrence of neurogenesis-to-gliogenesis switch depends on two distinct molecular events, the cessation of neurogenesis and the initiation of gliogenesis, with the latter process better characterized [43,47]. Over the past decades, several reports have indicated Sox9 as the master transcription factor mediating the switch to gliogenesis, specifying the glial fate choice through interacting with requisite partner factors like NF-1A and subsequently recruit key metabolic and migratory genes for initiation of astro-gliogenesis [43]. In addition, Notch1 and the JAK/STAT signaling cascade act in concert to trigger the temporal transition to gliogenesis by transactivating promoter of the glial-specific gene *Gfap* [47]*.* Conversely, molecular machinery that inhibits neurogenesis during the gliogenic phase remains largely elusive. Having observed that the number of VZ-derived neuronal subtypes increased remarkably only after E16.5 in the *Sox9* inactivated cerebellum, we suggest that this is due to a failure in the suppression of neuronal production at the VZ after the onset of gliogenesis. As discussed before, GABAergic interneurons are generated from specified progenitors in the prospective white matter (PWM) since late gestation. One may therefore argues that increased neurogenesis occurs at this secondary germinal site rather than within the neural progenitor pool at the VZ. While certainly possible, we believe that this is unlikely the case since we did not observe any increase in BrdU incorporation rate at the PWM since E16.5. Importantly, significant increase in the number of Pax2-expressing interneurons born at E16.5 was noticed in our *Sox9* mutants, suggesting the production of GABAerigc interneurons outside the PWM at late gestation. We also examined whether increased neurogenesis occur at a developmental stage prior to the onset of gliogenesis. Since no significant difference in the number of Pax2-expressing cells born at E13.5 between control and *Sox9-*null cerebellum was observed, this strongly supports that the surplus of GABAergic interneurons were born at E16.5 or thereafter. Taken together, our results indicate that *Sox9* inactivation in the cerebellum has elicited a prolonged period of neurogenesis at the VZ, which extended way beyond the normal neurogenic period and into the gliogenic phase. Our results therefore suggest *Sox9* can promote the progression of neuron-to-glial switch in cerebellum by suppressing the neural progenitors from adopting a neurogenic program. Indeed, various studies have demonstrated that while the neural progenitor pool becomes more biased to glial commitment as development proceeds, their neurogenic capacity is retained intrinsically [57,58], suggesting some molecular mechanisms must counteract the neurogenic capacity of the neural progenitors thereby prevent neurogenesis from occuring. Interestingly, the up-regulation of *Ngn2* expression at E16.5 probably reflects the abnormal neurogenic potential in *Sox9-*null neural progenitors after the neuron-glia switch. Notably, neurogenesis was extended at the expense of gliogenesis in the *Sox9*-ablated spinal cord. By contrast, gliogenesis proceeds normally in the cerebellum despite compromised suppression of neurogenesis in the absence of Sox9. Hence, we believe that the primary functional role of Sox9 in modulating the neuron-versus-glia switch is to suppress the neurogenic program of the neural progenitors, rather than actively triggering the onset of gliogenesis. In support of this view, overexpression of Sox9 in the adult subventricular zone (SVZ) cells completely abolished neuronal production, while the suppression of which by miR-124 is a critical step in promoting neurogenesis at the adult glial stem cell niche [15]. In contrast to this view are the previous findings showing Sox9 is the master regulator for gliogenesis and actively promotes glial lineage progression. It was proposed that Sox9 facilitates gliogenesis in a feedforward mechanism, by inducing NFIA expression and subsequently associates with the transcription factor, which together activates glial-specific genes [43]. However, normal glial specification in our mutants suggested that Sox9 is not absolutely critical for the formation of such a gliogenic-transcriptional complex. Since the inactivation of *Sox9* impaired the termination of neurogenesis in the entire CNS, *Sox9* is probably an instructive cue for timing of neurogenesis. On the basis of this primary function, *Sox9* also acts as a permissive factor for the onset of gliogenic program however, of which the importance and requirement of *Sox9* may be tissue/organ dependent. Taken together, our study provides novel evidence on the primary functional roles of Sox9 during neuron-glia fate switch in cerebellum, while challenges the current view of Sox9 as the key initiating factor for gliogenesis in CNS.

## Conclusion

*Sox9* is dispensable for neural stem cell induction, maintenance and multipotency in the cerebellum. Our results indicate that Sox9 mediate the neuron-to-glia fate switch by modulating the termination of neurogenesis, but not the initiation of gliogenesis as in other CNS compartments.

## Methods

### Generation of conditional knockout mice

The generation and genotyping of conditional (floxed) Sox9 (*Sox9*^*fx/fx*^) mouse line [59], *En1*^*Cre*^ [23], *Pax2*^*Cre*^ mouse line [22] and *ROSA26R* reporter mouse line [60] have been described previously. To generate conditional cerebellum specific *Sox9* knockout mutant, we crossed heterozygous either *En1*^*Cre*^ or *Pax2*^*Cre*^ mice with homozygous *Sox9*^*fx/fx*^ mice to generate *En1*^*Cre/+*^; *Sox9*^*fx/+*^ or *Pax2*^*Cre/+*^; *Sox9*^*fx/+*^ males. To obtain *En1*^*Cre/+*^; *Sox9*^*fx/fx*^ or *Pax2*^*Cre/+*^; *Sox9*^*fx/fx*^ mutant embryos for analysis, *Sox9*^*fx/fx*^ females were then crossed with either *En1*^*Cre/+*^; *Sox9*^*fx/+*^ or *Pax2*^*Cre/+*^; *Sox9*^*fx/+*^ males. Their respective *Sox9* heterozygous littermates with *Cre* allele, which developed normally, were used as controls. Since the pedigrees have been maintained by intercross breeding, the mouse genetic background is a mixture of C57BL/6 J, CD-1 and 129/Sv strains. Crosses were established and noon of the day when a vaginal plug was identified was designated as embryonic day (E) 0.5, while the day of birth was designated as postnatal day (P) 0. Mice bearing the wild-type, *floxed* and conditional null *Sox9* alleles, as well as the *Cre* and *ROSA26* reporter alleles were determined by PCR analyses using DNA extracted from the tails or yolk sac tissues as described previously [61]. At least three embryos for each genotype were examined in the experiments described below and embryos from the same litter were used for comparisons between the mutant and control. All animal procedures were conducted with the approval of the Animal Experimentation Ethics Committee of The Chinese University of Hong Kong.

### X-gal staining

E9.5 embryos were harvested in ice-cold PBS and fixed in freshly prepared 4% (w/v) paraformaldehyde (PFA) in PBS for 15 minutes at 4°C. After fixation, embryos were rinsed three times with Rinse Buffer [0.02% (v/v) NP40; 0.01% (v/v) deoxycholate; PBS] for 15 minutes at room temperature and then stained with Staining Solution [5 mM K_3_Fe(CN)_6_; 5 mM K_4_Fe(CN)_6_; 0.02% (v/v) NP40; 0.01% (v/v) deoxycholate; 2 mM MgCl_2_; 5 mM EGTA; PBS; 1 mg/ml X-gal] at 37°C overnight in dark. Following staining, embryos were post-fixed with 4% PFA in PBS at 4°C overnight.

### Immunohistochemistry

Timed-pregnant mice were sacrificed and the uterine horns were removed. Dissected brains from embryos were fixed in 4% (w/v) PFA in PBS at 4°C overnight. Fixed tissues were then dehydrated through an ethanol series, paraffin-embedded and sectioned at 7 μm thickness. For general histological examination, sections were mounted on Superfrost® Plus slides (Thermo Scientific) and stained with hematoxylin and eosin. For immunofluorescence staining, deparaffinized sections were subjected to antigen retrieval with heating in 10 mM sodium citrate buffer (pH 6.0) by microwave. Sections were blocked in 10% horse serum, incubated in primary and secondary antibodies for overnight at 4°C and 1 h at room temperature respectively. Nuclei were counterstained with Hoechst 33342 (1:1000, Molecular Probes). Sections were mounted in ProLong® Gold antifade reagent (Molecular Probes). For cryosections, brains were isolated from fetal mice and fixed in 4% PFA in PBS for 2 hours at 4°C. Tissues were then washed several times in PBS and cryoprotected in 20% (w/v), followed by 30% (w/v) sucrose in PBS and Tissue-Tek® OCT^TM^ compound (Sakura) for 1 night each at 4°C. The brains were then embedded in OCT medium in liquid nitrogen and stored at −80°C prior to sectioning. Tissues were sectioned at 10 μm thickness and stained as described above. Pictures were taken using either epifluorescence microscope equipped with a CCD camera (DP72; Olympus) or confocal scanning microscope (FV1000, Olympus).

### RNA extraction, reverse transcription and real-time PCR

Total RNAs were extracted from E18.5 cerebellum using TRIzol reagent (Invitrogen) following the manufacturer’s protocol. Reverse transcription was performed with 2 μg of extracted RNAs using the SuperScript III First-Strand Synthesis System (Invitrogen). Quantitative real-time PCR was performed with QuantiFast SYBR Green PCR Kit (Qiagen), transcript expression level was analyzed in triplicates using the comparative cycle time (*C*_*t*_) method and normalized with β-actin. Experimentally validated primers were adopted from PrimerBank [62] unless specified otherwise. Primers targeting *Sox8*, *Sox9* and *Sox10* were described previously [63]. Primers for β-actin were as follows: 5′-TAA AGA CCT CTA TGC CAA CAC AGT-3′; 5′-CAC GAT GGA GGG GCC GGA CTC ATC-3′.

### Bromodeoxyuridine incorporation assay

Pregnant mice were injected intraperitoneally twice at 30-minute intervals with bromodeoxyuridine (BrdU) dissolved in sterile saline at 100 μg/g body weight and then sacrificed 30 min after injection. For BrdU tracing experiments, injections were performed as described at E13.5 or E16.5 and embryos were harvested at E18.5. Embryos were fixed in 4% (w/v) PFA in PBS at 4°C overnight, embedded in paraffin and sectioned at 7 μm thickness. BrdU-labelled cells were revealed by immunostaining with anti-BrdU antibody as described above.

### Quantification of proliferating cells, cell count experiments and statistical analysis

Quantitative analysis of proliferating cells in the cerebellum were conducted by counting at least 10 alternative parasagittal paraffin sections (7 μm) from the cerebellar vermis to obtain the average labeling indices. To estimate the labeling indices at the VZ, the number of BrdU positive cells was divided by the total number of VZ cells for each section. To quantify proliferative cells in the prospective white matter, the number of BrdU immunoreactive cells was divided by the area of cerebellum analyzed using the ImageJ software version 1.46r (http://rsb.info.nih.gov/ij/). Quantifications for Purkinje cells and interneurons were performed by counting all Foxp2^+^ and Pax2^+^ cells in 10 equally spaced alternative sections from the cerebellar vermis. Statistical analyses for all cell count experiments were conducted using the GraphPad Prism 5 software (GraphPad Software Inc., San Diego, CA, USA). Data were expressed as means ± SEM. Student’s *t*-test was employed for group comparisons. Calculated *p*-values of less than 0.05 (*p* < 0.05) were considered as statistically significant.

### Antibodies

Primary antibodies and dilutions used were as follows: rabbit anti-BLBP (1:500, Millipore ABN14); rat anti-BrdU (1:70, Abcam ab6326); rabbit anti-Calbindin D-28K (1:250, Millipore AB1178); rabbit anti-cleaved caspase-3 (1:200, Cell Signaling Technology 9664); rabbit anti-EAAT1 (GLAST; 1:250, Abcam ab416); rabbit anti-Foxp2 (1:1000, Abcam ab16046); rabbit anti-Ki67 (1:100; Millipore AB9260), rabbit anti-Lim-1 (Lhx1; 1:100, Millipore AB3200); rabbit anti-Pax2 (1:500, Invitrogen 71–6000); rabbit anti-Pax6 (1:1000, Millipore AB2237); mouse anti-PCNA (1:200, Thermo Scientific MA5-11358); rabbit anti-Sox2 (1:200, Millipore AB5603); rabbit anti-Sox9 (1:2000, Millipore AB5535); goat anti-hSox9 (1:200, R&D Systems AF3075); goat anti-Sox10 (1:50, Santa Cruz sc-17342). For the secondary antibodies, HRP-conjugated affinity-purified goat anti-rabbit (Millipore AP132P) or anti-mouse IgG (Millipore AP308P) were used at a dilution of 1:250. Alexa Fluor®-conjugated secondary antibodies (Invitrogen A21245, A11024, A11055) were used at 1:1000.

## Abbreviations

BG: Bergmann glia
BLBP: Brain lipid binding protein
BrdU: Bromodeoxyuridine
CNS: Central nervous system
EAAT1: Excitatory amino acid transporter 1
GFAP: Glial fibrillary acidic protein
HMG: high mobility group
PBS: Phosphate-buffered saline
PC: Purkinje cell
PCL: Purkinje cell layer
PCNA: Proliferating cell nuclear antigen
PFA: Paraformaldehyde
PWM: Prospective white matter
RGC: Radial glial cell
RL: rhombic lip
VZ: Ventricular zone
